# How can global physical activity surveillance adapt to evolving physical activity guidelines? Needs, challenges and future directions

**DOI:** 10.1136/bjsports-2020-102621

**Published:** 2020-11-23

**Authors:** Richard P Troiano, Emmanuel Stamatakis, Fiona C Bull

**Affiliations:** 1 Risk Factor Assessment Branch, National Cancer Institute, Bethesda, Maryland, USA; 2 School of Health Sciences, Faculty of Medicine and Health, University of Sydney, Sydney, New South Wales, Australia; 3 Prevention on Noncommunicable Disease, Organisation mondiale de la Sante, Geneve, Switzerland; 4 School of Human Science, The University of Western Australia, Perth, Western Australia, Australia

**Keywords:** physical activity, surveillance, accelerometer

## Abstract

Public health guidelines on physical activity (PA) establish national policy agendas and provide the basis for setting goals and targets. Advances in measurement and resulting new scientific findings lead to evolution of PA guidelines. PA surveillance serves to track compliance with national guidelines, usually expressed as the proportion of the population ‘meeting’ the main quantitative guidelines. The WHO recently completed a process to review and update the global PA guidelines. Changes to the guidelines, such as removal of a 10-min bout criterion, pose challenges for PA surveillance. We review the evolution of PA guidelines and associated surveillance methods and explore implications of the updated guidelines for changes in population surveillance and opportunities for technological approaches to PA to enhance surveillance.

## Introduction

Public health guidelines on physical activity (PA) establish national policy agendas and provide the basis for setting goals and targets. PA is well established as a major contributor to health and well-being.^1 2^ Yet physical inactivity levels globally are a major public health concern.^1 3 4^

In conjunction with the release of the *Global Action Plan on Physical Activity 2018–2030: More Active People for a Healthier World*,^1^ and at the request of the World Health Assembly in 2018, the WHO recently completed a process to review and update the *Global Recommendations on Physical Activity for Health*,^5^ published in 2010. This involved an extensive review of the latest scientific evidence relating PA to health outcomes and led to recommended revisions to the guidelines. These updated guidelines are described elsewhere in this special issue.^6^ Such guidelines require national population-based surveillance systems to provide the mechanism of regular assessment and reporting.

Population surveillance is a core public health function to monitor priority health and disease indicators and their associated risk factors. Well-established national systems have regularly monitored diet, tobacco use and alcohol consumption for decades and yet only more recently has PA been included. Globally, this results in gaps in data, ad-hoc national or subnational surveys or countries with no data at all. Yet, since 2004, the WHO has recommended regular national surveillance of PA.^7^

The primary aim of monitoring PA is to track compliance with national guidelines, usually expressed as the proportion of the population ‘meeting’ the main quantitative guidelines.^8 9^ Changes to the PA guidelines can require changes in how PA is monitored, either in the instrument used and/or data analyses and reporting. These changes to national and global surveillance systems and indicators may over-ride the desire for instrument stability that supports tracking population compliance.

Assessing the implications of the updated WHO guidelines for population surveillance is a priority at both the national and international levels, as many countries, as well as WHO, value the comparability of data on PA over time and across countries. WHO has developed recommended tools for national PA surveillance systems, so the release of new global PA guidelines presents a catalyst to review current recommended assessment instruments and protocols for data reporting to inform and strengthen PA surveillance. This paper discusses the evolution of PA guidelines and the associated surveillance methods. We explore implications of the updated guidelines for changes in population surveillance and opportunities for technological approaches to PA to enhance surveillance. Our focus will be limited to the assessment methods for PA, including sedentary behaviour. Other critical aspects of population surveillance, such as sample design and statistical weighting to provide nationally representative estimates, recruitment methods to achieve high response rates and timely reporting, are outside the scope of this paper.

## Evolution of PA guidelines

Many countries have PA ‘guides’,^10^ ‘guidelines’^11^ or ‘recommendations’.^12^ These terms reflect a variety of purposes for the corresponding documents. They may be behavioural or programmatic guides for choices that will increase PA levels.^10^ Others synthesise available science and prescribe recommended amounts and types of PA.^11^ The documents may be clinical exercise guidance for health professionals to aid them in designing and implementing an exercise regimen for individuals,^13^ or consumer-friendly guides intended for the general public.^14^ For this paper, the term ‘guidelines’ refers to documents based on scientific evidence that prescribe recommended amounts, intensities and types of PA.

An overview of PA guideline evolution is presented in table 1. Early PA guidelines were promulgated by clinical professional organisations and focused on exercise to improve physical fitness and performance, which is purposeful, sustained and usually performed in specific locations. These features facilitated reporting the behaviour for monitoring. During the 1980s, the growing evidence on associations of PA with chronic disease prevention led to a paradigm shift from solely a clinical prescription to a broader public health approach for PA guidelines. Studies found benefits of PA carried out as part of activities of daily living, not necessarily as part of exercise training sessions, as well as activities that occurred in short episodes and were of moderate intensity, such as climbing the stairs or walking for transportation. This evidence supported a new set of guidelines that promoted the health benefits of moderate intensity PA that could be accumulated throughout the day by making active choices.

**Table 1:** Milestones in evolution of physical activity guidelines

Date	Example organisation(s) or countries	Focus	Targets	Selected features
1970s	American College of Sports Medicine (ACSM), American Heart Association 56 57	Increase fitness via exercise, minimise risk of adverse events	20 min, 3+times/week	Balance of endurance and muscle strength
Mid-1990s	Centres for Disease Control and Prevention (CDC)/ACSM, US Surgeon General^58 59^	Accumulate moderate-intensity PA to reduce non-communicable diseases (NCDs)	30 min of moderate-intensity aerobic most days of week	Minimal focus on muscle-strengthening
Early 2000s	US Department of Health and Human Services (HHS), WHO, Canada, Australia, other high-income countries (HICs)^5 16 17 60^	Accumulate moderate-intensity PA to reduce NCDs and improve quality of life (QoL)	150–300 min/week moderate-intensity or equivalent aerobic, muscle-strengthening 2+times/week	Increased focus on progress below target levels. ‘Some is better than none’.
2018–2020	US HHS, WHO, other HICs^11 21^	Accumulate moderate-intensity PA and reduce sedentary behaviour to reduce NCDs and improve QoL	150–300 min/week moderate-intensity or equivalent aerobic, muscle-strengthening 2+times/week	Increased emphasis on reducing sedentary behaviour; remove bout criterion

In the 1990s, the quantitative target was summarised as at least 30 min on most days (often quantified as 5 days) of the week, and brisk walking was the iconic example of appropriate intensity. Based on the available evidence, for activities to ‘count,’ they had to occur in bouts of at least 10-min duration. This new guideline approach complicated assessment of PA because routine sources of PA are more challenging to recall and quantify. Over time, this primary guideline for adults of 30 min of moderate-intensity to vigorous-intensity PA (MVPA) on 5 or more days per week was adopted in numerous national guidelines and became the basis of many PA surveillance activities.^15^

In the 2000s, PA guidelines evolved further. Responding to growing evidence of the health detriments of an increasingly sedentary lifestyle, guidelines from the USA and other nations and the WHO included ‘softer’ targets for PA below the MVPA thresholds. For example, the 2008 US PA Guidelines (2008 PAG) included a recommendation to ‘Avoid inactivity’ and stated that ‘Some activity is better than none’.^16^ Simultaneously, the target for adults became a weekly volume of 150–300 min per week of moderate-intensity aerobic activity or 75–150 min of vigorous intensity or an equivalent combination of the two intensities. The earlier recommendation of 30 min on 5 or more days per week was noted as one way to achieve this amount. Muscle-strengthening activities for all major muscle groups were recommended at least two times a week for all adults. Muscle strengthening was particularly emphasised for older adults, as was the inclusion of activities to improve balance for fall prevention. Other countries utilised the evidence base developed for the 2008 PAG to update their guidelines and achieved greater international harmonisation of recommendations.^5 17–19^ In many cases, countries were able to adapt their surveillance protocols to these new guidelines without changing their instruments by analysing and reporting weekly minutes of PA rather than a frequency of meeting 30 min on at least 5 days.

In 2018, the US Department of Health and Human Services updated their review of the scientific evidence and issued the *Physical Activity Guidelines for Americans*, second edition.^11^ Whereas studies of PA and chronic disease epidemiology shaped the paradigm shift towards public health guidelines of the 1990s, the growing availability of device-based data from cohorts with mortality and morbidity outcomes influenced the most recent evolution. Another significant development was the rapid growth in studies of sedentary behaviour. The quantitative recommendations for aerobic and muscle-strengthening PA remained unchanged. However, the recommendation on reducing sedentary behaviour was strengthened and simplified to ‘Move more and sit less’. While the evidence did not support a specific time limit for sedentary behaviours, these data did show that adverse effects of total sedentary time (most often measured as sitting) could be attenuated by increased amounts of MVPA.^20^

The further evolution of PA guidelines in response to scientific research advances on PA and health has led to three major challenges for surveillance, two current and one future. First, the elimination of a 10-min bout requirement results in a potential need for minute-by-minute measurement of activity intensity.^6 11 21^This is a profound challenge for standardised survey instruments. Second, youth activity recommendations have changed from recommending *at least 60* min per day to recommending *an average of 60* min per day.^6 21^This may require changes in survey questions and sampling regimens for surveillance. Finally, there is growing recognition that healthful activity recommendations require integrated attention to behaviours within the 24-hour day.^14 22 23^ The potential inclusion of sleep in future global guidelines would require substantial surveillance adjustments.

PA guidelines are based on evolving science, which means that the integration of PA surveillance and guidelines requires adaptation in an iterative process with changes in measurement leading to evidence for new guidelines that in turn require new measurement approaches for surveillance. Device data provided insights that shaped recent PA guidelines, but device-based guidelines are still years away. Current PA guidelines rely on evidence from studies with self-reported MVPA. However, emerging demand for measures of comprehensive PA from multiple activity domains places heavy demands on surveillance systems for PA. More cohorts with device-based measures and morbidity and mortality follow-up are needed to derive device-based guidelines. Therefore, at least for now, modification of self-report surveillance instruments is necessary. The next section addresses the evolution of PA surveillance, primarily through self-reports.

## Population surveillance of PA

Population-based health surveillance systems collect and report on key health indicators to inform policy development, programmatic priorities and provide a mechanism of accountability for policy implementation and progress.^8 9^ The inclusion of PA indicators within health surveillance systems has a comparatively shorter history than other non-communicable disease (NCD) risk factors such as blood pressure and tobacco use. This might be because WHO only launched the first global policy on PA in 2004^7^ and the first global guidelines on PA in 2010,^5^ but it may also reflect the complexity of assessing the multiple components of PA and practical constraints of surveillance systems.

The most common approach to assess PA is with self-report. Self-report methods have the advantage of being relatively inexpensive to administer, are generally unobtrusive and they can be adapted to different country contexts. To monitor compliance with PA recommendations, the surveillance instrument needs to assess multiple dimensions of PA behaviour. Quantifying volume of PA requires assessment of frequency (how often), duration (how long) and intensity (eg, moderate or vigorous). Additionally, assessing specific types of activity (aerobic or muscle-strengthening) is necessary, as these characteristics have independent health benefits. PA surveillance data and reporting has historically focused on aerobic activities because of strong evidence on the association of aerobic PA with reduced risk of coronary heart disease^24 25^ despite the inclusion of muscle strengthening in global and national PA guidelines.^26^ Details of the key dimensions of PA assessment are outlined in box 1.

Box 1 **Dimensions of physical activity and sedentary behaviour assessment**

Physical activity is a complex set of behaviours, with possible measurements made of its frequency, duration, intensity and activity type.

**Frequency** is how often physical activity is undertaken: measures of frequency are usually expressed in a defined time frame. The timeframe used can vary, for example, past week, usual week, usual weekday and weekend day, past 2 weeks, past month and some use past school term or over past year.

**Duration** of physical activity is usually expressed in hours and minutes and reported per session, or total time per day.

**Intensity** of physical activity can be based on self-perceived intensity (relative) or activities may be presented in categories and classified as light-intensity, moderate-intensity or vigorous-intensity based on assigned energy expenditure values in Metabolic Equivalent of Task that express intensity as a multiple of basal resting energy expenditure.^61 62^

**Type(s) of activity** can be classified by categories such as aerobic/cardiorespiratory, muscle or bone strengthening, balance or flexibility, although few types of physical activity are solely one type. Specific activities can also be documented directly by requesting respondent report, or the instrument can provide a checklist of activities (eg, swimming, running, cycling, walking).

**Domain or context** of activity can also be assessed by structuring the instrument using location and purposes of physical activity such as household tasks, occupational, transportation or leisure/recreation and sports.

**Sedentary behaviour** is often assessed as a standalone behaviour but increasingly more detailed approaches that assess different dimensions of sedentary behaviours are now available.^63^

Some early population surveillance instruments for PA in adults originated in epidemiological studies.^27^ However, each instrument approached PA differently, with various recall periods and response options. These variations limited comparability between population estimates and prompted the WHO, in collaboration with the CDC and Karolinska Institute, to convene the first global expert meeting to review the status of PA surveillance and research needs. An international collaboration developed and ultimately produced the International Physical Activity Questionnaire (IPAQ). Four versions were created that varied in length and detail (a short or long form) and administration mode (self-report/household interview or telephone interview). The short version was recommended for population surveillance and widescale adoption quickly followed in many countries as well as region-wide surveys, such EUROBAROMETER for European Union member and candidate countries.^28^

Although IPAQ short had many advantages over other contemporary instruments, its approach to collecting time spent in vigorous-intensity and moderate-intensity PA and walking without context was a limitation. Contextual cues in survey questions such as asking about walking *for transportation* can facilitate recall and contribute to measurement of total PA rather than focusing on leisure. Context also serves to connect surveillance to policy evaluation by measuring PA in different domains that may be related to inequalities in PA levels and might be influenced by specific policies.

The international PA community was interested in the observation of wide variation in sources of PA by domain across countries at different levels of economic development.^29^ To advance NCD surveillance, WHO developed a standardised system known as STEPwise approach to Surveillance (STEPS)^30^ that included PA. The Global Physical Activity Questionnaire (GPAQ) was developed by adapting the items and structure of IPAQ to address three domains: work, leisure and transport.^31^ Both IPAQ and GPAQ became commonly used by many countries, particularly low-income and middle-income countries (LMIC) that were establishing health surveillance systems. By 2016, GPAQ had been used in more than 100 countries and more than 50 countries had used IPAQ.^32^ Both instruments provide data to track compliance with the 2010 global PA guidelines.^5^ Most high-income countries (HIC) with well-established surveillance instruments retained their own country-specific instruments and methods. Neither IPAQ nor GPAQ has been updated since their development.

The development of PA surveillance among children and adolescents has primarily progressed through assessment tools and protocols for use in the school setting and focuses on adolescents (aged 11–17 years). The Health Behaviour in School-aged Children,^33^ mostly undertaken in Europe and North America, and the Global School-based Student Health Survey,^34^ covering the rest of the world, both include questions to report on meeting the 2010 WHO PA guidelines for youth.^5^ Major gaps remain in assessment of PA in younger school-aged children (under 10 years) and those under 5 years,^35^ despite the fact that global guidelines for youth include ages 6–10 years and guidelines now exist for children under the age of 5.^22^ These gaps clearly arise because younger children lack the cognitive skills needed to answer standardised survey questions about behaviour^36^ and are a further argument for accelerating progress in device-based measurement for PA surveillance.

Although global PA surveillance has progressed, gaps remain for monitoring PA among some subpopulations. This may be related to new guideline aspects that are not part of surveillance instruments, such as balance exercises for older adults, or due to small samples of populations that are included in guidelines, such as persons with disabilities. Trend data are also lacking in the majority of countries.^37^ Existing PA surveillance systems rely on self-reports that are recognised to have considerable amounts of measurement error.^38–40^ In the next section, we discuss the potential of digital technologies, particularly wearable devices, to assess PA in surveillance systems. By replacing self-report based on short instruments using standardised survey questions, devices would theoretically reduce measurement error. However, it is not obvious how they would help address gaps related to measurement of PA in the populations mentioned above.

### The potential for digital devices and technology to advance PA surveillance

The growing popularity of wearable trackers and fitness apps in recent years and the vast amounts of data that they generate present attractive possibilities for surveillance.^41^ This contributed to the recent WHO *Global Action Plan on Physical Activity 2018–2030* call for development and testing of digital technologies, including wearable devices to strengthen population PA surveillance.^1^ Wearable devices and other technological approaches to PA assessment remove much of the potential bias due to self-report and the cognitive challenges of recalling many routine behaviours. Devices also allow for more precise calculation of average daily PA if data are collected across multiple days. For population estimates, a single random day of measurement per person with adequate sample size provides valid estimates of group level PA.^42^ The challenges of self-report and related potential advantages of devices are amplified by the recent evolution of guidelines to remove the minimum 10-min bout requirement, emphasise total activity from all sources and intensities and recommend an average daily PA duration for youth.

Accelerometer-based devices have been used in epidemiological research and surveillance for approximately 20 years^43^ and pedometers for even longer. However, application of wearable technology in population PA surveillance has occurred in only a small number of HIC. Japan has a long history of tracking step-counts in the population with pedometers^44^ and Canada has repeated PA measures with accelerometers.^45 46^ The USA has twice utilised accelerometers in the National Health and Nutrition Examination Survey;^43 47^ other countries have also used pedometers or accelerometers in national surveys or representative subsamples (online supplementary table) and even performed pooled analyses across countries.^48^ Application in other settings, such as epidemiological cohorts, to supplement or replace self-report is becoming more widespread, but still mostly limited to HIC (online supplementary table).

10.1136/bjsports-2020-102621.supp1 Supplementary data

Wider scale adoption, particularly for population surveillance in both HIC as well as LMIC, remains limited by a number of methodological and practical challenges. First, before wearable devices can be used for PA surveillance a consensus on the interpretation of the resulting data is needed. Interpreting device-based data in relation to PA guidelines poses challenges due to the variations in translating the acceleration signals to PA behaviour information. For example, data from waist-worn devices that primarily measure ambulatory motion are typically interpreted with absolute intensity categories (eg, sedentary, light, moderate and vigorous). However, calibration studies have produced multiple different intensity cut-offs to define those categories.^49^ Second, practical issues remain unresolved, especially wear location, which affects what aspects of PA can be measured effectively. Major wear location sites include waist or hip, wrist and thigh. Each has advantages and disadvantages with waist and wrist wear most common in recent large studies (online supplementary table). For example, wrist wear improves compliance, but increases error associated with hand movement, while thigh wear allows estimation of posture,^50^ but estimates of intensity are limited to stepping cadence.^51^ Use of devices for PA surveillance will require greater efforts to calibrate data from different wear locations and inevitable compromises concerning which aspects of PA are best captured.

A third limitation for PA surveillance by devices is that across all single placements, stationary exercises (eg, yoga, strength training), and exercises involving limbs without monitors cannot be captured well. Therefore, no wearable device currently captures all required components of PA guidelines. For example, single accelerometer-based methods cannot capture required metrics (frequency, intensity and muscle groups) of muscle-strengthening exercise, which is a key component of the WHO,^5^ ^21^ as well as many national PA guidelines.^11^ The same limitation applies to the balance exercise component of guidelines^5 11^ ^21^ for older adults. Wear of multiple devices has the potential to capture most guideline components, but participant burden and resource demands are likely unacceptable for surveillance.

When considering the use of consumer-marketed wearable devices and the data they generate for surveillance, four more concerns arise. The first concern is data ownership. Device manufacturers may or may not be interested in sharing data with governments for public health surveillance. Furthermore, the users themselves may or may not be interested in sharing such data. A US-based survey found that only 40% of wearable users would be willing to share their data with a public health agency.^52^ Population representativeness is a second concern for use of wearable device data for surveillance. Despite the rapid growth in fitness tracker and application use, global population penetration is less than 5% and varies greatly by age and income.^53^

A third concern for surveillance is the short lifespan of wearable devices. Typically, no model stays in the market for more than 1–2 years. Required replacement of devices due to loss and failure will result in a mix of older and newer devices with unknown data comparability. Furthermore, and lastly, device manufacturers process data with proprietary algorithms. These algorithms are frequently updated with unknown effects on data comparability as software changes can alter the metrics obtained from a given level of activity.

Taken together, these concerns remain a significant barrier to adoption of device-based measurement for PA surveillance. Such devices hold great potential to improve the measurement of PA within surveillance systems, but for the coming decade, we will likely continue to rely largely on self-report for global surveillance of PA.

## Implications of new guidelines for PA surveillance

National guidelines on PA and sedentary behaviour set the framework for policy agendas and action. Progress in scientific understanding of the health effects of specific amounts and types of PA and developments in measurement that facilitate new insights have shaped the evolution of PA guidelines. As guidelines change, population surveillance system must adapt to provide relevant and useful information. Technological innovation has made wearable devices that assess PA and sedentary behaviour more available, practical and affordable, offering promising opportunities. Yet our brief overview of progress and challenges suggests that in most countries, devices are not ready to replace self-report instruments for PA surveillance. So the challenge remains to adapt self-report measures to align with the key changes made in the new global PA guidelines, namely the removal of the 10-min bout criterion for the adult recommendation on aerobic activity and the change from *at least* 60 min every day to an *average* of 60 min per day across a week in the youth recommendations.

A simple option to address the removal of the bout criterion is to remove language that specifies reporting only 10 min or longer bouts. This change would be expected to result in greater reported PA duration. However, reported amounts may be less than expected because respondents cannot accurately quantify incidental brief activity. Studies with and without 10-min bout criteria will be needed to examine the effect of any wording change. Inclusion of both types of question in existing surveillance systems would help to understand the effect of the change on trends.

In general, youth represent the most challenging area for PA surveillance.^36 54^ Adapting surveillance to the change in the youth recommendations may be accomplished by developing new questions and establishing a mechanism for transition or by retaining current questions in order to continue trend assessments and adding an additional question(s) to capture daily average PA as required by the new recommendation. However, changing question wording will not address the need for PA surveillance in children unable to answer complex questions about behaviour over the past days, weeks or months and use of proxy report may require multiple respondents including parents and teachers, both of whom miss observing large portions of the day. The alternative of requesting daily durations for a full week may be more accurate but increases survey response time. Measurement of daily PA is a strength of wearable devices that is employed by Canada to calculate average duration for youth.^55^

As noted previously, PA guideline development and related surveillance are in transition from evidence and guidelines based on self-report to potential development of device-based guidelines. We already see the contribution of device measures in the removal of the bout criterion and recognition of total volume of PA. Device measures from epidemiological studies continue to inform guideline development and will gain value as follow-up time for morbidity and mortality outcome increases. With further advances and consensus on translating accelerometer signals to behavioural measures relevant to PA guidelines as well as solutions to other practical challenges, digital devices may become feasible for PA surveillance.

Other challenges for the alignment of PA surveillance and guidelines relate to sedentary behaviour and PA types beyond aerobic. The updated WHO guidelines for sedentary behaviour are non-quantitative. Therefore, there is no way to assess who ‘meets the guidelines’. A specific threshold is elusive because the current evidence indicates that the detrimental effect of sedentary behaviour depends on the amount of MVPA. In contrast, muscle-strengthening PA has a frequency target of at least twice a week, but the recommendation also specifies ‘all major muscle groups’ and moderate or greater intensity, both of which present challenges for feasible surveillance.

Beyond surveillance for specific types of PA, surveillance needs to improve or be developed for population groups that now are specifically included in the PA guidelines. These populations include preschool children, pregnant and postpartum women, older adults and persons with disabilities or chronic conditions. Many current surveillance systems do not include preschool children, or even preadolescents. Although pregnant and postpartum women and persons with disabilities may not be excluded from surveillance, their numbers are likely to be too small for reasonable prevalence estimates. The guidelines for the other adult groups, such as older adults, differ from those of all adults in subtle ways by emphasis or adjustment of the general recommendations so pertinent questions need to be developed. At a minimum, these populations need to be included and identifiable in PA surveillance.

## Conclusions

National governments and international organisations develop PA guidelines to inform and support policy on PA and interconnected areas, such as sports, urban planning and active sustainable transportation. These policies and PA targets require monitoring, and national population-based surveillance systems provide the mechanism of regular assessment and reporting. Despite the challenges to surveillance that updating PA guidelines brings, they represent the best current science and should be adopted in national health policy and citizen guidance.

PA guideline development and related surveillance are currently in transition. Wearable devices have clearly advanced understanding of the relationship between PA and health. These data have spurred evolution in PA guidelines, but challenges remain that must be addressed before wearable devices will provide metrics for surveillance that are directly comparable to current PA guidelines. Currently, no single measurement modality can capture all desired metrics for PA surveillance. In the interim, as we try to monitor PA guidelines that still rely on evidence from self-report, wearable devices may be able to supplement traditional PA surveillance, but current self-report instruments need to be adapted to address the evolving PA guidelines.

**What is already known**

- Physical activity’s health benefits have led to development of national physical activity guidelines.
- Population surveillance provides a means to monitor compliance with guidelines.

**What are the new findings**

- Updated physical activity guidelines pose challenges to physical activity surveillance.
- Device-based measures may facilitate surveillance, but challenges remain.
