# Supplementary material for: How can global physical activity surveillance adapt to evolving physical activity guidelines? Needs, challenges and future directions
Source: Br J Sports Med. 2020 Nov 23;54(24):1468–73. doi: 10.1136/bjsports-2020-102621 (PMC7719905; doi:10.1136/bjsports-2020-102621)
Supplement: Supplementary data [file bjsports-2020-102621supp001.pdf]

Supplemental Table. Examples of nationally representative studies and large cohorts with device-measures

Survey or cohort	Dates	Participant ages	Device	Wear location/time	Approximate sample size
National Health and Nutrition Study of Japan [1]	Annually since 1989	15 and older (20 and older since 2012)	Pedometer Yamasa AS-200	Waist/waking	n ~ 3,500-4,000 per year
National Health and Nutrition Examination Survey (NHANES) [2,3]	2003-2006	6 and older	ActiGraph AM-7164	Waist/waking	n ~ 7,000 per cycle
	2011-2014	3 and older (ages 6 and older for 2011 only)	ActiGraph GT3X+	Non-dominant wrist/24-hours	
Portuguese Population Study [4]	2006-2008	10 and older	ActiGraph GT1M	Waist/waking	n ~ 4,700
Norwegian Children Study [5]	2005-2006	9 and 15 years	ActiGraph 7164	Waist/waking	n ~ 2,300
Canadian Health Measures Survey [6]	2007-2009<br>2009-2011<br>2012-2013<br>2014-2015<br>2015-2017	3-79 years (ages 6-79 for cycle 1 2007-2009)	Actical	Waist/waking	n ~ 5,000 per cycle
Health Survey for England [7]	2008	4 and older	ActiGraph GT1M	Waist/waking	n ~ 2,000
Australian National Nutrition and Physical Activity Survey (NNPAS) [8]	2011-2012	5 and older	Pedometer G sensor model 2026	Waist/waking	n ~ 6,000
Finnish Health 2011 Study [9]	2011-2012	18-85 years	Hookie AM1 20	Waist/waking	n ~ 1,500
Swedish Attitude, Behavior and Change (ABC) Study [10]	2002<br>2008	18-74 years	ActiGraph MTI (2002)<br>ActiGraph 7164 (2008)	Waist/waking	n ~ 1,100 and 500
Latin American Study of Nutrition and Health (ELANS)[11]	2014-2015	15-65 years	ActiGraph GT3X	Waist/waking	n ~ 2700
**Selected Large (n >1000) cohorts**					
European	1997-1998	9-15 years	MTI	Waist/waking	n ~ 4,000

Youth Heart Study (EYHS) [12]			ActiGraph 7164		6- and 12- year follow-up completed in certain countries
Avon Longitudinal Study of Parents and Children (ALSPAC) [13, 14]	2001-2009	Children:<br>11/12 years<br>13/14 years<br>15/16 years	ActiGraph AM 7164 or ActiGraph GTM1	Waist/waking	n ~ 6,000 children subsample
Personal and Environmental Associations with Children's Health (PEACH) [15]	2006-2008	10-11 years	ActiGraph GT1M	Waist/waking	n ~ 1,300
Child Heart and Health Study in England (CHASE) [16]	2006-2007	9-10 years	ActiGraph GT1M	Waist/waking	n ~ 2,000
Sport Physical Activity & Eating Behaviour: Environmental Determinants in Young People (SPEEDY) [17]	2007	9-10 years	ActiGraph GT1M	Waist/waking	n ~ 2,000
Identification and Prevention of Dietary- and Lifestyle-Induced Health Effects in Children and Infants (IDEFICS) [18]	2007-2008	2-11 years	ActiGraph ActiTrainer or GT1M	Waist/waking	n ~ 7,000
Millennium Cohort Study [19]	MCS4: 2008-2009	7 years	ActiGraph GT1M	Waist/ waking	n ~ 4,000
	MCS6: 2015-2016	14 years	GENEActiv	Wrist/24-hours	
Activity and Function of the Elderly in Ulm (ActiFE Ulm) [20]	2009-2010	65 and older	ActivPAL	Thigh/24-hours	n ~ 1,200

REasons for Geographic and Racial Differences in Stroke (REGARDS) ancillary study [21]	2009-2013	black and white participants, 45 and older	Actical	Waist/waking	n ~ 8,000
Western Australian Pregnancy Cohort (Raine) Study [22]	2011	22-23 years	ActiGraph GT3X+ x 2	Waist + wrist/24-hours	1989 Birth cohort n ~ 2,000 Year 22: n ~ 900
International Study of Childhood Obesity, Lifestyle and the Environment (ISCOLE) [23]	2011-2013	9-11 years	ActiGraph GT3X+	Waist/24-hours	n ~ 7,000
Pelotas Birth Cohorts[24]	2004 Cohort 2010	~6 years	GENEActiv	Wrist/24-hours	n ~ 2,600
	1993 Cohort 2011	~18 years	GENEActiv	Wrist/24-hours	n ~3,600
	1982 Cohort 2012-13	~30 years	GENEActiv	Wrist/24-hours	n ~2,700
Women's Health Initiative – Objective Physical Activity and Cardiovascular Health ancillary study (OPACH) [25]	2012-2014	women 63 and older	ActiGraph GT3X+	Waist/waking	n ~ 7,000
German National Cohort Study [26]	2013-2019	20-69 years	ActiGraph GT3X+	Waist/24-hours	n ~ 200,000
Tromsø Study [27]	Tromsø 7: 2015-2016	40 and older	ActiGraph wGT3X-BT	Waist/24-hours	n ~ 6,300
UK Biobank [28]	2013-2015	45-79 years	Axivity AX3	Wrist/24-hours	n ~ 100,000
Nord-Trøndelag Health Study (HUNT) 4 [29]	2017-19	13 and older	Axivity AX3 x 2	Thigh + lower back/24-hours	n ~ 38,000

REFERENCES

1. Katanoda K, Matsumura Y. National Nutrition Survey in Japan. *Journal of nutritional science and vitaminology* 2002;48(5):423-32.
2. Troiano RP, Berrigan D, Dodd KW, et al. Physical activity in the United States measured by accelerometer. *Med Sci Sports Exerc* 2008;40(1):181-8. doi: 10.1249/mss.0b013e31815a51b3 [published Online First: 2007/12/20]
3. Troiano RP, McClain JJ, Brychta RJ, et al. Evolution of accelerometer methods for physical activity research. *Br J Sports Med* 2014;48(13):1019-23. doi: 10.1136/bjsports-2014-093546 [published Online First: 2014/05/02]
4. Baptista F, Santos DA, Silva AM, et al. Prevalence of the Portuguese population attaining sufficient physical activity. *Med Sci Sports Exerc* 2012;44(3):466-73. doi: 10.1249/MSS.0b013e318230e441 [published Online First: 2011/08/17]
5. Kolle E, Steene-Johannessen J, Andersen LB, et al. Objectively assessed physical activity and aerobic fitness in a population-based sample of Norwegian 9- and 15-year-olds. *Scand J Med Sci Sports* 2010;20(1):e41-7. doi: 10.1111/j.1600-0838.2009.00892.x [published Online First: 2009/05/09]
6. Tremblay M, Wolfson M, Gorber SC. Canadian Health Measures Survey: rationale, background and overview. *Health Reports* 2007;18:7-20.
7. Scholes S, Coombs N, Pedisic Z, et al. Age- and sex-specific criterion validity of the health survey for England Physical Activity and Sedentary Behavior Assessment Questionnaire as compared with accelerometry. *Am J Epidemiol* 2014;179(12):1493-502. doi: 10.1093/aje/kwu087 [published Online First: 2014/05/28]
8. Australian Bureau of Statistics. 4364.0.55.004 - Australian Health Survey: Users' Guide, 2011–13 2013 [Available from: https://www.abs.gov.au/AUSSTATS/abs@.nsf/Lookup/4363.0.55.001Main+Features12011-13?OpenDocument accessed 2 May 2020.
9. Husu P, Suni J, Vähä-Ypyä H, et al. Objectively measured sedentary behavior and physical activity in a sample of Finnish adults: a cross-sectional study. *BMC Public Health* 2016;16(1):920. doi: 10.1186/s12889-016-3591-y
10. Hagströmer M, Kwak L, Oja P, et al. A 6 year longitudinal study of accelerometer-measured physical activity and sedentary time in Swedish adults. *Journal of Science and Medicine in Sport* 2015;18(5):553-57. doi: https://doi.org/10.1016/j.jsams.2014.07.012
11. Ferrari GLM, Kovalskys I, Fisberg M, et al. Socio-demographic patterning of objectively measured physical activity and sedentary behaviours in eight Latin American countries: Findings from the ELANS study. *Eur J Sport Sci* 2019:1-12. doi: 10.1080/17461391.2019.1678671 [published Online First: 2019/10/12]
12. Riddoch C, Edwards D, Page A, et al. The European Youth Heart Study—Cardiovascular Disease Risk Factors in Children: Rationale, Aims, Study Design, and Validation of Methods. *Journal of Physical Activity and Health* 2005;2(1):115-29. doi: 10.1123/jpah.2.1.115
13. Mattocks C, Ness A, Leary S, et al. Use of Accelerometers in a Large Field-Based Study of Children: Protocols, Design Issues, and Effects on Precision. *Journal of Physical Activity and Health* 2008;5(s1):S98-S111. doi: 10.1123/jpah.5.s1.s98
14. Riddoch CJ, Leary SD, Ness AR, et al. Prospective associations between objective measures of physical activity and fat mass in 12-14 year old children: the Avon Longitudinal Study

of Parents and Children (ALSPAC). *BMJ* 2009;339(nov26 2):b4544-b44. doi: 10.1136/bmj.b4544

15. Page AS, Cooper AR, Griew P, et al. Independent mobility in relation to weekday and weekend physical activity in children aged 10–11 years: The PEACH Project. *International Journal of Behavioral Nutrition and Physical Activity* 2009;6(1):2. doi: 10.1186/1479-5868-6-2
16. Owen CG, Nightingale CM, Rudnicka AR, et al. Ethnic and gender differences in physical activity levels among 9-10-year-old children of white European, South Asian and African-Caribbean origin: the Child Heart Health Study in England (CHASE Study). 2009;38(4):1082-93. doi: 10.1093/ije/dyp176
17. Van Sluijs EM, Skidmore PM, Mwanza K, et al. Physical activity and dietary behaviour in a population-based sample of British 10-year old children: the SPEEDY study (Sport, Physical activity and Eating behaviour: Environmental Determinants in Young people). *BMC Public Health* 2008;8(1):388. doi: 10.1186/1471-2458-8-388
18. Konstabel K, Veidebaum T, Verbestel V, et al. Objectively measured physical activity in European children: the IDEFICS study. *International Journal of Obesity* 2014;38(S2):S135-S43. doi: 10.1038/ijo.2014.144
19. Gilbert E, Conolly A, Tietz S, et al. Measuring young people's physical activity using accelerometers in the UK Millennium Cohort Study. *Centre for Longitudinal Studies Working Paper* 2017
20. Denkinger MD, Franke S, Rapp K, et al. Accelerometer-based physical activity in a large observational cohort-study protocol and design of the activity and function of the elderly in Ulm (ActiFE Ulm) study. *BMC geriatrics* 2010;10(1):50.
21. Howard VJ, Rhodes JD, Mosher A, et al. Obtaining Accelerometer Data in a National Cohort of Black and White Adults. *Medicine & Science in Sports & Exercise* 2015;47(7):1531-37. doi: 10.1249/mss.0000000000000549
22. Straker LM, Hall GL, Mountain J, et al. Rationale, design and methods for the 22 year follow-up of the Western Australian Pregnancy Cohort (Raine) Study. *BMC Public Health* 2015;15(1) doi: 10.1186/s12889-015-1944-6
23. Katzmarzyk PT, Barreira TV, Broyles ST, et al. The International Study of Childhood Obesity, Lifestyle and the Environment (ISCOLE): design and methods. *BMC Public Health* 2013;13(1):900. doi: 10.1186/1471-2458-13-900
24. Ricardo LIC, Wendt A, Galliano LM, et al. Number of days required to estimate physical activity constructs objectively measured in different age groups: Findings from three Brazilian (Pelotas) population-based birth cohorts. *PLoS One* 2020;15(1):e0216017. doi: 10.1371/journal.pone.0216017 [published Online First: 2020/01/11]
25. Lacroix AZ, Rillamas-Sun E, Buchner D, et al. The Objective Physical Activity and Cardiovascular Disease Health in Older Women (OPACH) Study. *BMC Public Health* 2017;17(1) doi: 10.1186/s12889-017-4065-6
26. German National Cohort (GNC) Consortium. The German National Cohort: aims, study design and organization. *European Journal of Epidemiology* 2014;29(5):371-82. doi: 10.1007/s10654-014-9890-7
27. Sagelv EH, Ekelund U, Pedersen S, et al. Physical activity levels in adults and elderly from triaxial and uniaxial accelerometry. The Tromsø Study. *PLOS ONE* 2019;14(12):e0225670. doi: 10.1371/journal.pone.0225670

28. Doherty A, Jackson D, Hammerla N, et al. Large Scale Population Assessment of Physical Activity Using Wrist Worn Accelerometers: The UK Biobank Study. *PLOS ONE* 2017;12(2):e0169649. doi: 10.1371/journal.pone.0169649
29. Nord-Trøndelag Health Study. The Nord-Trøndelag Health Study. HUNT4 [Available from: https://www.ntnu.edu/hunt4 accessed 2 May 2020.
